# Abstract of the Proceedings of the Buffalo Medical Association

**Published:** 1858-06

**Authors:** Austin Flint


ART. II.—*Abstract of the Proceedings of the Buffalo Medical Association.*

TUESDAY EVENING, March. 2, 1858.

[The following cases were presented at the March meeting of the association, but were omitted in the report of that month owing to lack of space in our pages.]

*Prof. Hamilton* presented a specimen of

*Biliary Calculus, Cholesterine, and Bile Pigment; also small Calculi of Cholestine.*

Dr. Hamilton presented a specimen of biliary calculus sent to him by Dr. Harvey Jewett, of Canandaigua. It had been discharged from a lady sixty years old, who had passed previously a large number of smaller size. After the escape of this, she had no more trouble and is now perfectly well.

The specimen, six-eighths of an inch in its longest diameter, and five-eights in its shortest, has three plane and polished surfaces, or facets, indicating plainly that three other calculi were lodged with it. It is formed of concentric layers, of an alternate white and yellow, or yellowish red color. It weighs 50 grains.

Dr. Austin Flint, Jr., subjected the specimens to examination, by dissolving a portion of the calculus in boiling alcohol, filtering it while hot, and allowing it to cool; he discovered under the microscope, the characteristic tablets of cholesterine in the precipitate. Bile pigment also enters largely into its composition; indeed it is probably composed of alternate layers of both, the bile pigment occasioning the yellowish color, and the cholesterine the white.

*Prof. Hamilton* also presented three specimens of probably pure cholesterine, found in the gall bladder of a cow. They are white, translucent, and have a soft, soapy feel. Two of them are of the size of pepper corns, and the third is of the size of a small bean, oblong and irregular upon its surface.

Dr. Flint, Jr., had also examined these specimens, which contrary to his expectation, he found did not contain as much cholesterine as the other calculus, there being only a trace present, and most of the substance of the calculus being insoluble in boiling alcohol. It was tested for nothing but the above mentioned substance.

*Prof. Hamilton* also presented a specimen of

*Cancer of Uterus. Dilatation of Ureter, &c.*

This is an ordinary example of cancer of the uterus, occurring in a lady about forty years of age. The disease was only remarkable in the rapidity of its progress. She was residing at Fort Wayne, Indiana, when the occurrence of irregular and painful menstruation, with frequent hæmorrhages, announced the commencement of the malady. She consulted Dr. Hamilton about the last of Dec., 1857. Dr. H. found the neck of the uterus enlarged and presenting the characteristic signs of cancer. The husband was immediately apprized of the nature of the disease, and advised to return home as soon as possible.

Dr. B. S. Woodworth, of Wayne, her family physician, who had always regarded the disease as cancer, had the charge of Mrs. M. from this time until her death, which occurred about three weeks since. She did not survive the malady more than five or six months. Dr. W. sent the uterus and also one of the kidneys to Dr. Hamilton, which were laid before the society.

The cancerous degeneration involves the neck of the uterus and a large portion of its body.

One of the ureters and the pelvis of the kidney are greatly enlarged, probably as the result of obstruction occasioned by the mechanical pressure of the uterus.

*Prof. Hamilton* presented a case of

*Extirpation of the Eye, and Death after Six Years. Autopsy.*

Miss C. H., of West Bloomfield, Wyoming Co, N. Y., æt. 16 years, called upon Dr. Hamilton, June 12, 1852. Her health was good; her complexion and hair light. She has never seen at all with her right eye, not even to distinguish daylight from darkness; yet the pupil of this eye dilated and contracted as frequently as the pupil of the sound eye. The right eye was always a little smaller than the opposite; never has been painful.

About one year before, a tumor had begun to show itself at the upper and outer angle of the socket. At this time it was quite large, elastic, and large veins lay over its surface; the eye protruded one inch, and the conjunctiva was highly vascular.

*Operation.* Patient under influence of ether and chloroform. An incision was made through the upper lid, extending from one angle of the eye to the other, and carried deeper until the tumor was exposed. It was firmly attached to the periosteum covering the orbital plate of the frontal bone, and the mass was removed by peeling up the periosteum.

Beyond this, several distinct masses of diseased tissue, of a lobular form, were founb lying in the bottom of the orbit and surrounding the optic nerve in such a manner that it was impossible to remove them without destroying the nerve. It was immediately determined, therefore, to extirpate the eye and clean the socket entirely. This was accomplished chiefly by the scissors, the periosteum being subsequently torn from every point to which the tumors were attached. The bleeding was free, but it was easily restrained by pledgets of lint pressed into the socket until it was full. The lachrymal gland was removed with the eye.

The wound was closed with sutures, and dressed with lint, &c. During a part of the operation she was completely insensible; and at other times she would complain that some one was pulling her hair, probably when the supra orbital nerve was touched. After the operation she was faint, and vomited freely.

*Examination of the Product.* The lachrymal gland was enlarged to more than twice its natural size; its form was lobulated, and portions of its interior were hard and crispy, like cartilage, and portions soft and granular. The other masses were of a similar structure.

The outer tunics of the eye itself were sound, but a vertical section of the eye disclosed a curious growth from the retina. This was a pyramid, of a firm consistence and white color, with its base six lines in diameter, and perfectly round, resting on the retina, and its apex, truncated, and about one line in diameter, resting against the posterior surface of the lens. The lens was perfectly opaque. On cutting the pyramidal growth, the base appeared fibrous, and the anterior portion more solid, like cartilage.

The microscope detected no cancer cells.

The socket filled up rapidly, and the wound healed kindly, and for many years Miss H. continued to enjoy good health.

By a letter received from Dr. Harvey Jewett, of Canandaigua, Dr. Hamilton learns that Miss H. died somewhere about the tenth of last month; and although Dr. Jewett has given no account of the circumstances preceding her death, (he promises to furnish it soon for the Buff. Med. Journ.,) he has sent to Dr. Hamilton a portion of the meninges of the brain with certain morbid growths attached. These Dr. Hamilton showed to the society.

*First.* There are two tumors situated underneath the tentorium and resting upon the cerebellum, one of which weighs two ounces, and the other half an ounce. Both were attached by narrow pedicles to the dura mater, over and upon the margin of the meatus anditorius internus. They have evidently grown from the outer, or fibrous layer of the dura mater, or have been deposited between it and its serous surface, since the latter is continued over them throughout. There were slight vascular connections on the surface of the larger one but none on the smaller.

On cutting into these tumors, they seemed to be chiefly composed of a substance resembling the white portions of the brain, being soft, and homogenous; but a small part of each tumor was more solid, slightly vascular, and appeared to the naked eye, fibrous.

*Second.* Nearly the whole of the upper half of the dura mater, and especially that part near the superior longitudinal sinus and along the falx cerebri, was studded with deposits of a soft cerebriform substance, always underneath the serous surface, and of sizes varying from a split pea, to one inch in diameter.

Whether these deposits, or any portion of them, were tubercular, it would not be easy to determine, since the specimen had lain for some time in alcohol; yet this supposition is not improbable. It is probable, however, that such was not the character of the whole of the larger growths, or tumors; nor did Dr. Hamilton see any reason to suppose that the original tumors in the eye which he had removed six years before, were tubercular; indeed he is quite certain that they were not.

It is worthy of notice that the original tumors removed by Dr. H. from the orbit, seemed to grow from the periosteum, being firmly attached to it, and that these tumors found within the skull are attached likewise to the dura mater, or internal periosteum.

TUESDAY EVENING, April 6th, 1858.

The Association met.

Present—The President, Prof. Flint, in the chair; Drs. Hamilton, Eastman, Rochester, Lemon, King, Wilcox, Root, Gay, Newman, Nichell, White, Mixer, Miner, Ring, and Flint, Jr.

The minutes of the last meeting were not present, and on motion, their reading was dispensed with.

On motion, the election of officers, which was next in order, was suspended for a hearing of the report of officers for the past year.

*Dr. Newman* then read the report of the officers for the past year, which on motion, was accepted.

The report of the Treasurer was as follows:

| | |
|---|---|
| Balance in treasury, April, 1857, . . . . | $ 7 78 |
| Receipts during the year, . . . . . . | 173 71 |
| | 181 49 |
| Expenses during the year, . . . . . . | 179 80 |
| Balance in treasury, . . . . . . . | 1 69 |

The association then proceeded to an election, and the following officers were chosen for the ensuing year:

| | |
|---|---|
| *President*, . . . . | Dr. Wyckoff. |
| *Vice-President*, . . . . | " Newman. |
| *Secretary*, . . . . | " Flint, Jr. |
| *Treasurer*, . . . . . | " Mixer. |
| *Librarian*, . . . . | " Lemon. |
| *Primary Board*, . . . . | Drs. Hawley, Eastman, King. |

Moved and seconded, that the address of the retiring President be deferred until the next regular meeting. Carried.

Moved and seconded, that a committee of two be appointed to conduct the newly elected President to the chair. Carried; and the chair appointed Drs. White and Wilcox such committee.

The President, Dr. Wyckoff, then took the chair with a few appropriate remarks.

Moved and seconded, that a vote of thanks be tendered to the retiring President, for the able and impartial manner in which he had presided over the Association, and also that a similar vote be tendered to the Secretary, Treasurer, and other retiring officers, for the able and faithful discharge of their duties for the past year. Carried.

Moved and seconded, that the discussion of Dr. Newman's report on Puerperal Convulsions, be deferred until the next meeting, after its publication in the Buffalo Medical Journal. Carried.

*Prof. Rochester*, committee on Tracheotomy in Croup, made a partial report, which was received, and on motion, the committee was continued.

*Prof. Hamilton* then presented to the association the following specimen: An erectile tumor, containing a number of phlebolites. This was the first tumor of the kind which had ever come under his observation. It was situated on the arm of a woman 45 years of age, and was produced twenty-six years before by the rupture of a vein, caused by lifting a pail. When seen by Dr. H., it was about the size of a hen's egg; its surface irregular, elastic, and of a bluish color, looking like a varicose tumor; there were no other varicose veins in the arm, but there was a small varicose tumor at the inner angle of the right eye which, she says, has existed since childhood, and which is probably congenital. This is also blue and elastic, and about the size of a small fibert.

After removal, the tumor was found to contain a quantity of phlebolites; the specimen, when presented to the association, contained some of these phlebolites, which could be distinctly felt.

The phlebolites were submitted to Dr. A. Flint, Jr., for microscopic examination, who found them to consist of concentric layers of fibrin, which constituted the greater part of the bulk, enclosing a round, polished, and hardened mass, composed of fibrin and calcareous matter. This calcareous matter was apparent under the microscope, but had no crystaline structure. There were thirty or forty of these little bodies in various parts of the tumor, varying in size from a millet seed to a common pea.

The next was a specimen of a fleshy polypus of the nose. These growths are extremely obstinate, and Prof. Hamilton has never before successfully removed them. The patient was a lad æt. 16, who nine months ago, had his attention first called to this difficulty by hæmorrhage from the nose; about three months ago, his father brought him to Prof. H., who, finding it impracticable to remove it by torsion, tied the polypus with a wire ligature; and at the end of ten days it sloughed off during the night, and was swallowed; it was then about the size of a small pear.

On the first of March, 1858, the patient again called upon Prof. Hamilton, who found that it was growing rapidly, and endeavored to tear it away, expecting to plug the nares if the bleeding was very profuse, but he was unable to detach it by any amount of force which he could apply; he then applied the wire ligature as before, and allowed it to remain. On the third day, however, he succeeded in extirpating it by means of a strong pair of curved forceps introduced through the mouth into the posterior nares. The evulsion seemed to be complete, and was followed by very little hæmorrhage.

The tumor was about the size of a hen's egg, firm and fleshy in consistence. Some months have now elapsed and yet there is no return, and Dr. H. has every reason to believe that the lad is permanently cured.

*Prof. Hamilton* also presented a specimen of enlarged cervical glands about twenty in number. The patient was a lady, aged abut 50. Twenty years ago, a single gland became enlarged and was removed twelve years ago by Prof. James Webster. It was removed at that time, because it had begun to encroach upon the trachea and impede respiration.

Prof. Hamilton removed them March 26th, 1858. As before stated, there were about twenty distinct tumors, varying in size from a pea to a hen's egg; they seemed to contain tuberculous matter.

There seemed to Prof. Hamilton but two adequate reasons for removing enlarged lymphatics in this situation. One would be on account of their encroaching upon the trachea and impeding respiration. Another would be the development of a cancerous tendency. This latter occurrence may take place, though as a rule cancer commences in the secretory glands. Prof. Hamilton is sure that he has seen instances of this kind, some of the circumstances which would point to this tendency and which existed in this case, are, return if they have ever been extirpated before, and slow growth with no disposition to ulceration. Prof. H. is opposed to their removal on any other grounds. If removed, other glands are likely to become speedily affected in the same way.*

*Dr. Gay* reported the result of a case of extensive injury to the forearm, which was reported to the association some months since, by Dr. A. Flint, Jr. In this case the forearm was denuded of integuments from the elbow to the wrist, and about three-fourths of the radius was carried away. It was seen in consultation by Prof. Hamilton and Dr. Flint, Jr., and treated with dressings of cold water and simple cerate. In five months the ulcer was entirely healed, but the arm was exceedingly deformed and bent.

*Prof. Hamilton* remarked that he thought this an exceedingly instructive case, especially to the young practitioner; that he had never seen a case which promised less towards saving the limb. In this case he thought the recovery due to the extensive loss of integument, thereby allowing a free exit of fluids. When this was not the case there was more danger, though

* In making up his abstract of the proceedings, the secretary has made use of notes of the foregoing cases, which were kindly supplied to him by Messrs. Demainville and Mackey, students of medicine in the office of Prof. Hamilton.

at first sight the limb looked much better. Prof. H. had seen cases illustrating both of these points.

*Dr. Flint, Jr.*, remarked that it was a matter of which the profession of Buffalo could be proud; that such limbs as the one referred to, were saved by our surgeons. One of the greatest improvements in surgery, is the saving of limbs, which formerly would inevitably have been sacrificed. None in this country excelled Prof. Hamilton in this respect. Dr. F. had an opportunity of observing the practice of some of the most eminent surgeons in the country, during his stay in Philadelphia, and is confident that in this case, most of them would have amputated immediately; yet the result shows how much better was the course pursued by the advice of Dr. Hamilton.

*Prof. Hamilton* gave a sketch of a plastic operation which he had lately performed at the hospital. This was a case of elcoplasty. Prof. H. had made three operations of this kind, all of them being successful. This was a most unpropitious case; it was emphatically an old ulcer, which the Dr. had observed in the hospital for some time. The operation was made by sliding in a portion of new integument. When the ulcer first healed, the patient was attacked with hæmoptysis, and it was supposed that a more formidable disease had been substituted for the original difficulty; but this apprehension is not now entertained. The other two were cases at the hospital, of old standing. The operations were made two months ago. They are now nearly healed. Dr. Hamilton thinks that there can be no doubt as to the propriety of the operation, as there is certainly a limit to the possibility of reproduction of skin. In those cases it is necessary to make a new nucleus for the regeneration of integument, by transplantation.

*Prof. White* presented a new form of perforator. The instrument was designed by M. Hippolyte Blot, and was made to the order of Prof. W. by Tieman, of New York. Prof W. had always found a difficulty with the old form of scissors, in being compelled to use both hands in opening them, while it was desirable to have the finger on the head of the child. The peculiarity in this instrument was that the blades could be opened with one hand, which he considered a great improvement. There was also another improvement which he had suggested, namely a shoulder to prevent the cutting part of the instrument from going beyond the skull, as this is the only part which it is necessary to cut in the operation. If the operation of perforating the head be decided upon, it is desirable that it be done thoroughly, as it would be extremely awkward for the physician and painful to the friends, to see a child born living and mutilated. This, however, has occurred.

*Prof. White* also wished to mention a case of the return of an inverted uterus of long standing; he merely mentioned the case at that time, intending to communicate a more elaborate account of it hereafter. He had reported a case some time since, where a uterus was returned by him, after it had been inverted for eight days; but in this case it had been inverted for nearly *six months.* He received a communication from the attending physician at Hornellsville, Dr. C. D. Robinson, relative to the propriety of extirpation, as the patient was very much reduced by repeated and constant hæmorrhages. Prof. W. replied that he thought restoration possible, and at the patient's request, went to Hornellsville, and succeeded in returning it. He has since received a communication from the attending physician, giving the most favorable reports of the state of the patient, and he has every reason to expect a full recovery.

*Prof. Hamilton* accorded extraordinary credit to Prof. White for his enduring faith in the possibility of reduction, which has of course been denied by all authorities, and the perseverance with which he continued his efforts until the end was accomplished; and since the report of this case he thinks that there is no reason to suppose that a uterus cannot be returned even after a much longer period than six months; unless it had formed attachments in its new position.

*Dr. Miner* inquired if chloroform was administered during the operation.

*Prof. White* replied that he had not intended to give a full report of the case at present. Chloroform was used. The uterus had evidently finished the processes of involution and was no larger by actual measurement, than is usual in women who have borne children. The organ was completely returned, and the danger to the patient resulted from her extreme debility from loss of blood. We all know that the mouth of the uterus can be artificially dilated at any time, for the performance of operations in its interior, and why cannot the os be as well dilated by the pulling open of the lips by the vagina, after the uterus has been inverted?

*Prof. Rochester* presented a sketch of the following case:

On the fourth of March, he was called to a case of labor. The labor was rapid, and followed by alarming uterine hæmorrhage, which however, he succeeded in controlling. The patient then was doing well for three weeks. At 7, A. M., she was as comfortable as usual; but a few minutes after, was seized with a sudden and alarming hæmorrhage, although she had not yet got up. Prof. Rochester saw her at eight, when she was pulseless and apparently moribund; stimulants were immediately exhibited and a tampon introduced; by these means the hæmorrhage was controlled. In twenty-four hours the tampon was removed (the whole of it, as was supposed,) in an extremely offensive condition, and replaced by another saturated with a solution of alum. Prof. R. noticed that he could not introduce so large a tampon on this occasion, as at first, which he attributed to the contraction of the parts. In twenty-four hours more, the second tampon was removed, which was also exceedingly offensive. A discharge from the parts continued, and with strangury and symptoms of cystitis. Injections of the chlorinate of soda were made use of, in consequence of the offensive nature of the discharge, which was attributed to the character of the secretions. Five days after the removal of the second tampon, the nurse discovered something protruding from the vagina, which proved to be a piece of tampon, more than a yard of which was removed. During all this time, no critical examination of the vagina and os uteri was made, as the Dr. feared that the disturbance might reproduce hæmorrhage.

*Prof. White* called to mind a case of flooding which occurred more than four weeks after delivery; the patient was doing well before its occurrence; this patient died, although the tampon was used with every other means that could be employed for arresting the hæmorrhage and sustaining the vital forces.

*Dr. Lemon* wished to add his testimony to the case reported by Dr. White, where the muriated tincture of iron was used externally by mistake, with good effect. He had lately treated a case with good results, by its external application.

*Prof. Hamilton* remarked that cases of cure of any disease, which might be self-limited, should be stated as such, with a great deal of caution. He thought in such cases, it was difficult to ascribe any certain efficacy to any particular remedy.

Moved and seconded, that the treasurer be authorized to put up shelves for the reception of books and pathological specimens, at an expense not exceeding five dollars. Carried.

Moved and seconded, that the president be permitted to rent the small room belonging to the Association, at his discretion. Carried.

The Association then adjourned.

Friday Evening, May 7th, 1858.

The Association met as usual on the first Tuesday of the month, but adjourned to date.

Present—The President, Dr. Wyckoff, in the chair; Drs. Ring, Treat, Newman, Rockester, Rankin, Miner, and Hutchins.

Minutes of last meeting not present.

*Prof. Rochester*, the committee on Tracheotomy in Croup, then finished his report.

Moved and seconded, that the report be accepted, and ordered to be published. Carried.

(This report will appear in full in the next number of the Journal.)

*Prof. Rochester* moved that Dr. Bernard Monahan be invited to meet with the Association until he could become a member. Seconded and carried.

*Dr. Flint, Jr.*, moved that a similar invitation be extended to Dr. Stevens. Seconded and carried.

A letter was then read from Dr. Mixer resigning his office of treasurer of the association.

On motion, the resignation was accepted, and the association proceeded to ballot for a new treasurer. A ballot was taken, and Dr. Hutchins was duly elected.

The Association then adjourned.

AUSTIN FLINT, Jr., M. D.,
Secretary.

